# Rebreeding of Female Mountain Lions After Litter Mortality

**DOI:** 10.1002/ece3.73416

**Published:** 2026-04-13

**Authors:** Mark A. Peyton, Brian D. Jansen, James W. Cain, Jonathan A. Jenks

**Affiliations:** ^1^ Department of Biology New Mexico State University Las Cruces New Mexico USA; ^2^ Arizona Game and Fish Department Phoenix Arizona USA; ^3^ Department of Rangeland, Wildlife and Fisheries Management Texas A&M University College Station Texas USA; ^4^ Department of Wildlife and Fisheries Sciences South Dakota State University Brookings South Dakota USA

**Keywords:** cougar, den, infanticide, puma, reproduction

## Abstract

Despite litter size and kitten survival in mountain lions ( *Puma concolor* ) being well‐documented, there is a critical gap in the literature regarding the time that elapses between litter mortality and subsequent rebreeding in females. Here, we present observations from seven female mountain lions from two distinct study locations—the Black Hills of South Dakota and the Jemez Mountains of north‐central New Mexico—where female mountain lions lost litters and rebred shortly afterward. Our findings contribute novel data to the understanding of reproduction in mountain lions, including the shortest documented interval between litter loss and rebreeding (range: ≤ 4–94 days; mean: 46 days). These observations underscore the evolutionary pressures shaping sexual selection in mountain lions and highlight the significant physiological toll females endure as a consequence of infanticide and intraguild competition, with implications for population dynamics and fitness.

## Graphical abstract

We documented rapid rebreeding by female mountain lions ( *Puma concolor* ) following litter loss, including the shortest known rebreeding interval to date. These observations highlight the importance of infanticide and intraguild competition in shaping female fitness and population dynamics.

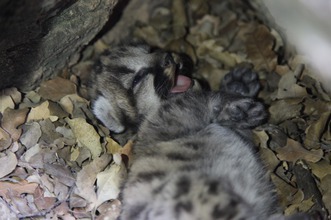

## 1 Introduction

Sexually selected infanticide is a reproductive strategy and evolutionary pressure observed in several large carnivores, where males kill offspring they did not sire to gain a reproductive advantage (Ebensperger 1998; Hrdy 1979). By eliminating unrelated young, males can accelerate the return to estrus in females, thereby increasing their chances of mating and maximizing their genetic contribution to the population (Ebensperger 1998; Palombit 2015). For example, by eliminating unrelated young, male lions ( *Panthera leo* ) were able to effectively shorten interbirth intervals for female lions, thereby maximizing their genetic contribution to the population (Pusey and Packer 1994). Although advantageous to males, this behavior imposes significant costs on females, who lose their reproductive investment and must endure the physiological demands of repeated reproduction.

To reduce the threat of infanticide, female carnivores have evolved several counterstrategies to enhance offspring survival and protect their reproductive investment, though these adaptations often come with their own risks and costs (Agrell et al. 1998; Ebensperger 1998; Palombit 2015). For example, females may mate with multiple males during a single estrus cycle to obscure paternity (Benson et al. 2012; Stasiukynas et al. 2022). While this counterstrategy reduces the likelihood of male aggression towards the offspring it also increases the risks of physical harm to the female during mating, limits her selection of genes for her offspring, and increases energy loss through additional breeding and resources lost (e.g., food) to the new mate (Agrell et al. 1998). Similarly, avoiding territories occupied by known infanticidal males may limit access to optimal foraging areas, impacting the female's ability to sustain herself and her young. In some cases, females actively defend their offspring, however, this behavior carries a high risk of injury or death to the female (Singh et al. 2014). Despite these female counterstrategies (Ebensperger 1998), infanticide remains a persistent threat, and the physiological and energetic costs of countering it can significantly reduce female fitness (Palombit 2015). The influence of these dual pressures—not only the risk of infanticide itself but also the demands of avoiding it—extend beyond individual reproductive success, to also shape the social structure and dynamics of populations where infanticide is prevalent.

Female carnivores with altricial young face compounding challenges of interspecific competition and intraguild predation of offspring (Polis 1981; Polis et al. 1989). In multipredator ecosystems, juveniles of subdominant carnivore species face heightened predation risk from sympatric predators; additionally, while not directly engaged in resource competition, their development and survival are indirectly constrained by the energetic and nutritional costs imposed on their mothers through interspecific competition. For example, juvenile cheetahs ( *Acinonyx jubatus* ) experience high predation rates from lions and hyenas ( *Crocuta crocuta* ), which heavily influence female maternal strategies, including den selection and cub‐rearing behaviors (Laurenson 1994). However, interspecific competition introduces additional risks beyond direct predation, such as resource displacement and increased energetic costs for females that must evade dominant competitors or find safer but less resource‐rich habitats (Jackson et al. 2014). This competition can reduce prey availability, prolong maternal foraging trips, and increase exposure to predators. The combined pressures of sexually selected infanticide, interspecific competition, and intraguild predation create a complex landscape of threats, forcing females to balance their own survival with strategies to protect their offspring.

The mountain lion ( *Puma concolor* ), also known as the cougar or puma, is a subdominant apex carnivore inhabiting a diverse range of ecosystems across the Americas (Sunquist and Sunquist 2002). Like many felids, mountain lions are considered induced ovulators with several short breeding events to stimulate ovulation occurring daily from 1 to 16 days (Quigley and Hornocker 2010; Sunquist and Sunquist 2002). Following a gestation period of approximately 92 days, females give birth to a litter of 1 to 6 altricial kittens, with an average litter size typically ranging from 2 to 3 (Sunquist and Sunquist 2002). Mountain lion kittens require ~8 weeks of development before they are capable of traveling with the adult female and begin consuming meat (Beier et al. 1995). During this first 8 weeks, the adult female will keep her offspring concealed in a den site, providing the kittens protection from predation and the weather (Beier et al. 1995; Elbroch et al. 2015). As a solitary species, female mountain lions raise young alone and will spend 70%–80% of their life in the company of dependent offspring (Sunquist and Sunquist 2002). Among the primary sources of mortality for mountain lion kittens are infanticide by adult males and intraguild predation by larger predators, such as wolves ( *Canis lupus* ) and bears (*Ursus* spp.) (Hornocker et al. 2009; Logan and Sweanor 2001; Ruth et al. 2011; Sunquist and Sunquist 2002). These pressures influence the reproductive strategies of female mountain lions, who may adopt countermeasures such as mating with multiple males to obscure paternity, relocating dens to avoid detection, or defending their offspring when faced with direct threats (Logan and Sweanor 2001).

One mechanism through which females may respond to these selective pressures is variation in maternal investment. Experienced females often demonstrate more effective prey acquisition, den site selection, and spatial strategies that reduce encounters with infanticidal males or competing carnivores (Engebretsen et al. 2024; Logan and Sweanor 2001). Increased maternal investment has been associated with higher kitten survival, suggesting that behavioral competence improves reproductive outcomes across successive litters (Engebretsen et al. 2024). In socially dynamic systems where male turnover or interspecific interactions elevate kitten mortality risk, inexperienced females may be disproportionately vulnerable to litter loss, both because of lower provisioning efficiency and reduced ability to anticipate or avoid social threats. Thus, variation in maternal investment may partially buffer the reproductive costs imposed by competitive pressures or prey scarcity (Engebretsen et al. 2024; Balme et al. 2017).

Concurrently with competitive pressures, reproductive success is also strongly shaped by prey availability. Across much of their range, mountain lion parturition peaks during spring and early summer, coinciding with pulses of vulnerable ungulate neonates that provide high energetic returns to lactating females (Bartareau 2020; Jansen and Jenks 2012; Ruth et al. 2011). Kittens typically reach independence between 12 and 24 months and sexual maturity between 2 and 4 years of age (Bartareau 2020; Robinette et al. 1961; Sweanor et al. 2000). Because maternal care extends for over a year, females that successfully rear litters to independence exhibit longer interbirth intervals, whereas females that lose litters may return to estrus within weeks to months, substantially shortening birth intervals (Bartareau 2020; Logan and Sweanor 2001; Rabb 1959; Seidensticker et al. 1973).

Litter loss can shift the timing of subsequent estrus and parturition, sometimes resulting in rapid rebreeding during periods of lower prey availability or increased environmental stress. Therefore, the consequences of mountain lion litter loss may extend beyond the immediate death of the offspring, creating cascading effects on subsequent reproductive cycles (Jansen and Jenks 2012). For the adult female, these repeated reproductive attempts may limit restoration of maternal body condition, elevate energetic strain, and increase vulnerability to injury or mortality while attempting predation on risky prey, creating a trade‐off of current versus future fecundity (Balme et al. 2017; Clutton‐Brock et al. 1989; Hudson et al. 2022; Mendl 1994). While kittens conceived after a previous litter loss are more likely to be born at times of reduced resource availability, leading to earlier dispersal and smaller body sizes (Jansen and Jenks 2012). These suboptimal conditions can impair their ability to compete for resources and increase their vulnerability during the critical sub‐adult dispersal phase. Thus, infanticide and interspecific killing act as powerful selective pressures, shaping maternal strategies, offspring development, and subadult strategies, with broad ecological implications.

Here, we report our observations of the conditions under which litter loss occurred and the subsequent interval between litter loss and rebreeding in female mountain lions across two distinct ecosystems: the Black Hills of South Dakota and the Jemez Mountains of New Mexico. Understanding these challenges provides critical insight into the reproductive ecology of this species and the broader evolutionary forces shaping their behavior.

## 2 Methods

Black Hills study area—From 2002 to 2009, we studied mountain lions in the Black Hills Ecoregion (14,600 km^2^), a landscape of ridges, valleys, and canyons surrounded by the Northern Great Plains. The area has a continental climate with summer temperatures ranging from −6°C to 39°C and winter temperatures from −25°C to 21°C, and an annual average precipitation of 45 cm and is dominated by ponderosa pine forests, with some spruce, aspen, and birch (National Oceanic and Atmospheric Administration 2025). Prey species included deer (*Odocoileus* spp.), elk ( *Cervus canadensis* ), bison ( *Bison bison* ), mountain goat ( *Oreamnos americanus* ), bighorn sheep ( *Ovis canadensis* ), and domestic livestock. Medium‐sized sympatric carnivores included bobcats ( *Lynx rufus* ) and coyotes ( *Canis latrans* ), with no other large carnivores present.

Mountain lions > 3 months old were captured using trained dogs, snares, traps, or free‐darting methods and immobilized with a telazol‐xylazine mixture, counteracted with yohimbine at recommended doses consistent with (Kreeger 1996). We estimated age of mountain lions using a combination of tooth wear and pelage characteristics, as well as noted any previous reproductive activity (Anderson and Lindzey 2000). While kittens < 3 months old were captured by hand and restrained in burlap sacks. Radio‐collars (MOD‐500 or expandable MOD‐125) were fitted on individuals > 3 weeks old. All procedures adhered to guidelines from the American Society of Mammalogists and were approved by South Dakota State University's Institutional Animal Care and Use Committee (Approval No. 07‐A024).

Jemez Mountains study area—From 2014 to 2024, we studied mountain lions in the Jemez Mountains of north‐central New Mexico as part of a broader landscape restoration effort on lands managed by Santa Fe National Forest and National Park Service (Bandelier National Monument and Valles Caldera National Preserve). The area is a mix of plateaus, peaks, and canyons, formed by historic volcanic eruptions. Vegetation varies with elevation, with dominant spruce‐fir forests and montane grasslands at the highest elevations descending to ponderosa pine and juniper woodland at lower elevations. The area has a semi‐arid high desert climate, with summer temperatures ranging from 23.8°C to 29.4°C and winter temperatures from −13°C to 10.5°C (Bennett et al. 2020; Bruggeman and Waight 2021). Annual precipitation averages 42 cm, with about 60% falling during the late summer North American Monsoon weather pattern (Bennett et al. 2020; Bruggeman and Waight 2021). Prey species primarily included mule deer (*Odocoileus hemionus*) and elk ( *Cervus canadensis* ). Medium‐sized sympatric carnivores included bobcats ( *Lynx rufus* ) and coyotes ( *Canis latrans* ), and American black bears ( *Ursus americanus* ) were the dominant large carnivore on the landscape.

Adult and subadult mountain lions were captured using Aldrich‐type nonlethal leg snares (Elbroch et al. 2013; Logan et al. 1999), and chemically immobilized using 2.0 mg/kg ketamine +0.075 mg/kg medetomidine and reversed with 0.3 mg/kg atipamezole (Kreeger et al. 2023). We estimated the age of mountain lions using a combination of tooth wear and pelage (Heffelfinger 2010). We estimated the weight of mountain lions from an equation utilizing body measurements achieved during capture (Jansen and Jenks 2011). Adults were fitted with iridium GPS collars (Vectronic Aerospace [Berlin, Germany]), programmed to achieve a GPS fix every 3 h and upload collar data every 24 h. Kittens were captured by hand and restrained in burlap sacks. Capture protocols followed acceptable methods (Sikes and the Animal Care and Use Committee of the American Society of Mammalogists 2016) and were approved by the New Mexico State University Institutional Animal Care and Use Committee (Protocols #2011‐038, 2015‐022, 2021‐013, 2303000252) and the National Park Service Institutional Animal Care and Use Committee (Protocol ID – NM_BAND.VALL_Cain_LargeMammal_2021.A2).

## 3 Field Observations

### 3.1 Black Hills

On 13 June 2007, we captured a litter of 3 (1 M, 2 F) kittens that were born to a radio‐collared adult female; age of the female was estimated at 5–6 years old. The condition of the kittens indicated that they were young; eyes were only half‐open and still appeared cloudy, legs were unsteady while standing, and inner ears were closed. We aged these kittens at 7 days old (Currier 1983). We outfitted the kittens with small, expandable radio‐collars (approx. 77 g, MOD‐125, Telonics, Mesa, Arizona), ear tags, and a unique tattoo. On 15 July 2007, a radio signal from one collar indicated a lack of movement and we investigated the collar's status. We located the collar 700 m from the original den site and observed 1 male kitten (approx. 41 days old), lying underneath a small pile of leaf litter and debris, apparently covered by the mother. There was no internal or external trauma evident and very small invertebrate larvae were present, suggesting the mortality was about 24 h old.

Three days after documenting the mortality we relocated the two surviving kittens 1.3 km from the mortality site in a pile of large rocks and recaptured them while the mother was absent. Upon recapturing these two kittens, we noted no external trauma or apparent ill‐effects from the radiocollars, which indicated proper fitting. On 23 July 2007, 5 days following recapture, we detected both radiocollars indicating another mortality event 2.3 km from the previous capture location. We immediately investigated the scene and discovered the remains of both kittens about 50 m apart. Both kittens had been fully consumed and only a small portion of the face and tip of tail remained with the collars. These kittens were about 49 days old on the day of death.

After 4 months of continued aerial telemetry, we again detected denning activity of the mother 4.4 km from the last mortality site. When we investigated the suspected location, we discovered two male kittens that were captured and estimated to be 21 days old. Back‐dating from the capture date, placed birth date of these kittens at 14 November 2007. Gestation periods for mountain lions average 92 days (Anderson 1983; Logan and Sweanor 2001; Quigley and Hornocker 2010) and 114 days elapsed from the estimated date of death of the first litter, which suggests that she rebred in as few as 22 days post mortality of the first litter of kittens.

### 3.2 Jemez Mountains

We captured a total of 38 mountain lions (19 females and 19 males) between 2016 and 2024. During this time, six females lost litters and subsequently rebred, with four of the females losing multiple litters (Table 1). We confirmed rebreeding at 4–94 days following the loss of a litter, contrasted to 333–631 days when at least one kitten survived to dispersal age (Table 1). Infanticide by unrelated male mountain lions was the leading cause of litter loss for females (6 of 11 litter mortalities); while litter abandonment (*n* = 2), black bear predation (*n* = 1), infanticide by a female mountain lion (*n* = 1), and unknown cause of death (*n* = 1) were also documented. Below we provide a detailed narrative of each litter mortality event and subsequent rebreeding by the adult female.

**TABLE 1:** Summary of mountain lion ( *Puma concolor* ) breeding observations and litter fate in Jemez Mountains, New Mexico from 2016 to 2024.

Individual ID	Days with male	Days between traveling with male and den initiation	Fate of litter	Estimated age of litter (days)	Days until rebreeding
F11	13	76	Male mountain lion	100	94
F11	6	83	Female mountain lion	31	NA
F16	3	84	Black bear	12	30
F16	13	84	Male mountain lion	3	77
F21	3	84	Abandonment	12	1–4
F43	Unknown	Unknown	Male mountain lion	NA	35
F25	Unknown	~97	Abandonment	11	50
F25	8	88	Unknown fatality	7	26
F25	Unknown	~94	Male mountain lion	4	10
F29	2	103	Male mountain lion	16	88
F29	8	91	Male mountain lion	15	NA
F21	8	86	Independence	> 365	NA
F23	Unknown	~94	Independence	> 365	612
F25	2	90	Independence	> 365	333
F25	2	91	Independence	> 365	NA
F26	Unknown	Unknown	Independence	> 365	534
F26	10	85	Independence	> 365	470
F26	3	NA	NA	NA	NA
F30	Unknown	Unknown	Independence	> 365	631
F30	4	93	Independence	> 365	NA
F39	7	97	NA	NA	NA
F43	1	90	Independence	> 365	NA

**FIGURE 1:**
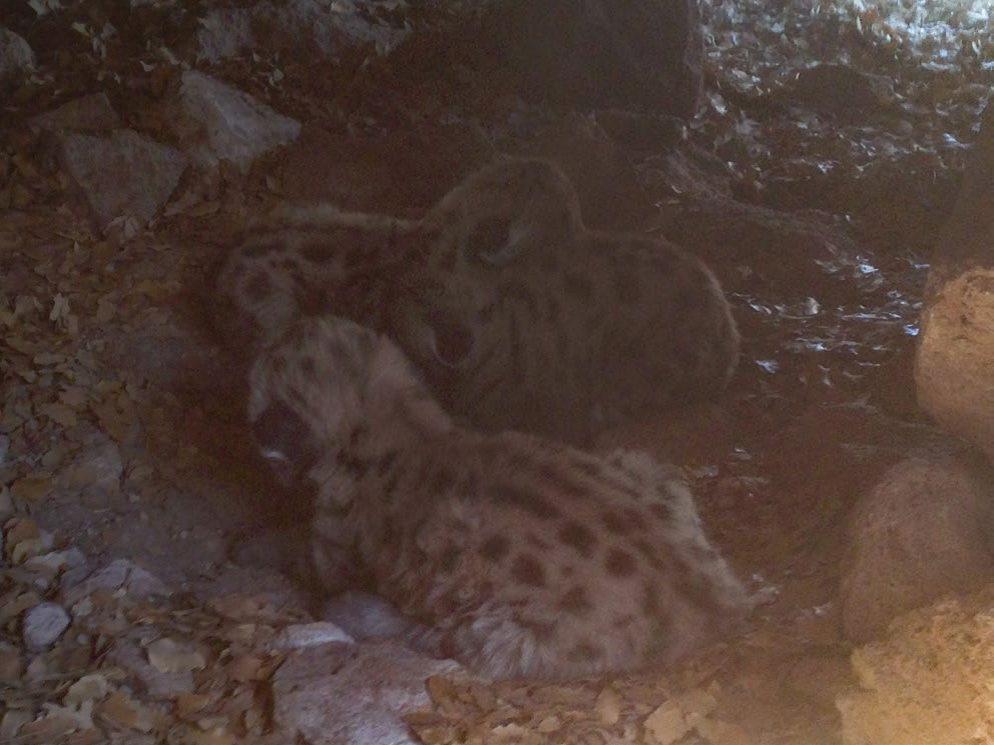
First litter of kittens identified for female 1 (F11) in the Jemez Mountains, New Mexico. A litter of 3 ~ 7‐day‐old kittens (2 male, 1 female) confirmed on 27 October 2016. Photograph has been lightened to improve visibility of kittens.

**FIGURE 2:**
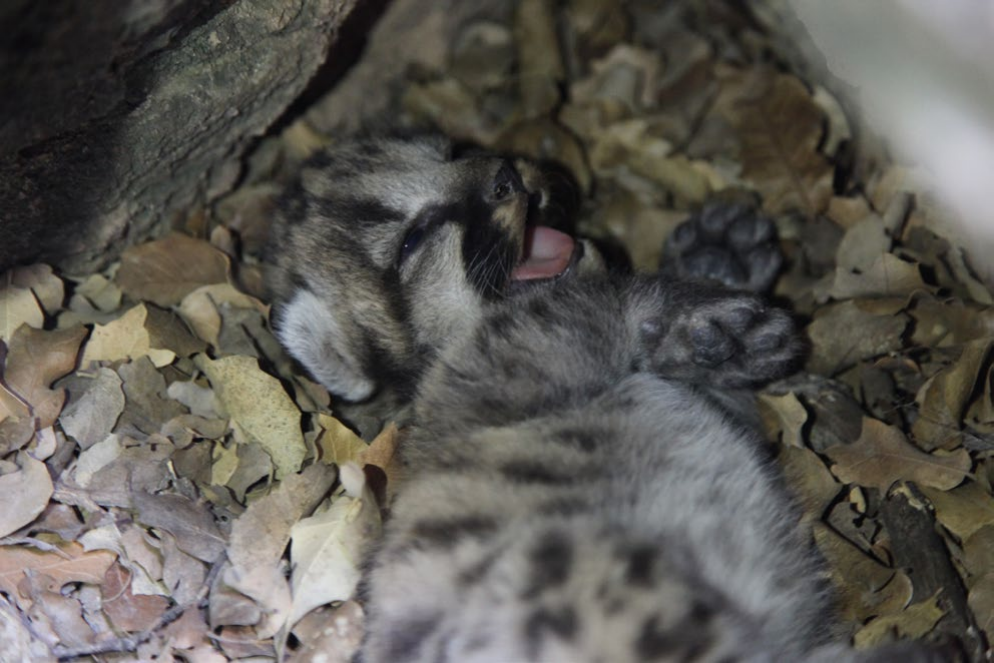
Second litter confirmed for female 1 (F11) in the Jemez Mountains, New Mexico. A single ~2 week old male kitten confirmed on 13 August 2017.

Female 1 (F11) – We captured a 5 year old, 36.3 kg adult female mountain lion on 20 July 2016 and observed her traveling with a collared male (M12) from 23 July 2016 to 5 August 2016. On 20 October 2016, her GPS collar indicated denning activity, and on 27 October 2016, we confirmed she had three kittens estimated to be < 2 weeks old (Figure 1). On 2 February 2017, the kittens were found killed and partially consumed by an uncollared male mountain lion. The kittens were estimated to have been killed within 24 h of identification and were estimated to be 100 days old at time of death. Subsequent GPS and camera trap data revealed that the female continued to visit the site and vocalized daily for over a week (Video 1). Between 27 and 31 March 2017 she rotated feeding at a kill site with the collared male she previously bred with in 2016 (M12); however, no breeding was observed on camera. Between 2 and 4 May 2017, GPS data identified that she was traveling with a different collared male (M08), and between 5 and 8 May 2017, she again traveled with the collared male she “shared” a kill with in March 2017, and bred with in 2016 (M12). One of these encounters in early May led to successful breeding, and she gave birth to a single male kitten by 13 August 2017 (Figure 2). Despite maternal care, the kitten appeared slightly lethargic and malnourished. Foraging behavior of the mother during development and feeding of the second litter was limited to small animals (< 15 kg), with no large (> 15 kg) kills documented.

**VIDEO 1:** Video of female 1 (F11) returning and vocalizing following the mortality of her first identified litter of kittens in the Jemez Mountains, New Mexico. Video content can be viewed at https://onlinelibrary.wiley.com/doi/10.1002/ece3.73416.

Between August and September 2017, the female moved the kitten to several secondary dens. On 24 September 2017, a different radio collared female (F16) was observed on camera visiting one of the inactive den sites in the same boulder field within ~100 m distance of the kitten. Unfortunately, due to the female (F11) frequently moving her kitten, we were unable to directly monitor the active den site to confirm if there was an interaction. However, despite repeated camera trap images of both adult females, the kitten was not observed again following this event. In the months following, the female resumed interaction with males, including at least one overlap with an uncollared male in October 2017 which lasted multiple days. The status of breeding during this overlap is unknown; however, no additional litters were documented before her harvest in February 2018. Key observations indicate a 94 day interval between her first litter loss and successful rebreeding, and 26 days between the loss of her second litter and association with another male; however, no kittens were identified following this interaction.

**FIGURE 3:**
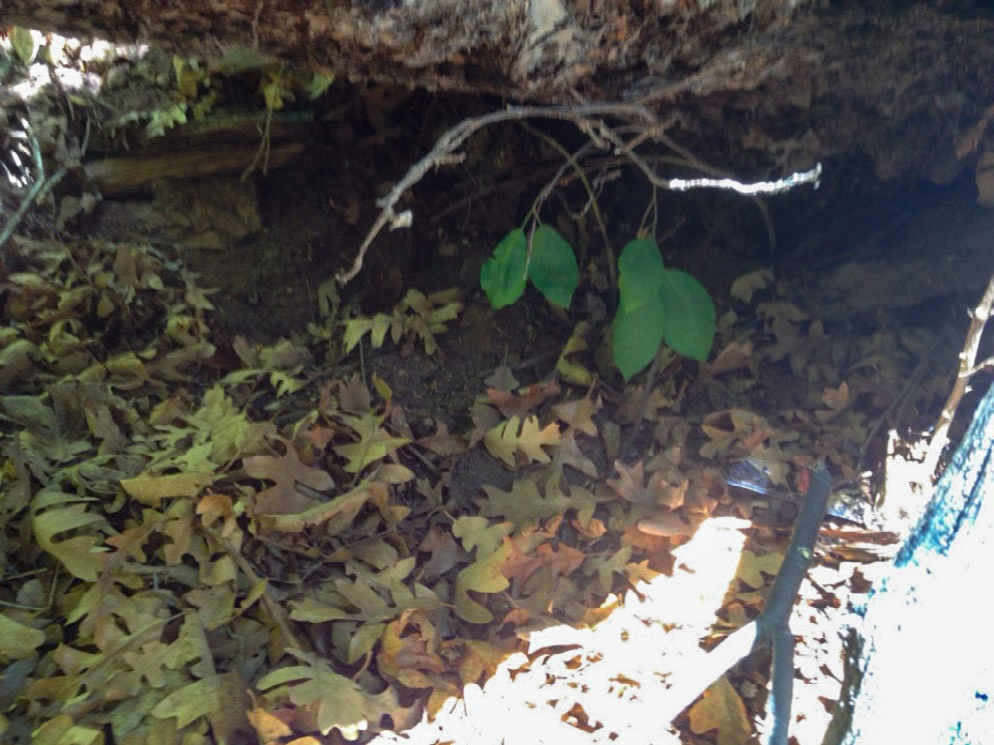
Photograph of confirmed den site for female 2 (F16). Photograph taken following kitten fatality.

Female 2 (F16) – On 16 March 2017 we captured a 37.6 kg adult female estimated to be 5–6 years old. Between 12 and 16 April 2017 her GPS data indicated she was traveling with a collared male (M10). On 14 July 2017 her GPS data indicated she initiated denning (Figure 3). On 20 July 2017 we confirmed ≥ 2 kittens; however, due to the presence of the female at the den we were unable to complete a thorough search or identify the sex of the kittens. On 29 July 2017 the kittens were killed by a black bear. On 29 August 2017 while visiting a GPS “cluster” we observed the female breeding with the same collared male (M10). Their GPS data indicates that the two traveled together from 28 August–10 September 2017. On 9 December 2017 we visited a suspected den site; however, there was no sign of kittens. Upon revisiting camera traps placed at previous kill sites for the female, we identified an uncollared male mountain lion at two of the female's sites between 30 November and 7 December 2017. We suspect this male is why we were unable to positively confirm kittens following the previous breeding event. Between 21 and 28 February 2018 we identified the female again traveling with an uncollared male; however, the female died in March 2018, before an additional litter could be confirmed.

GPS data combined with our observations confirm 30 days between the loss of the first litter and subsequent re‐breeding. We suspect this rebreeding event yielded kittens; however, we were unable to confirm this. If an undetected litter did occur, an additional ~82 days passed before rebreeding was again detected.

**FIGURE 4:**
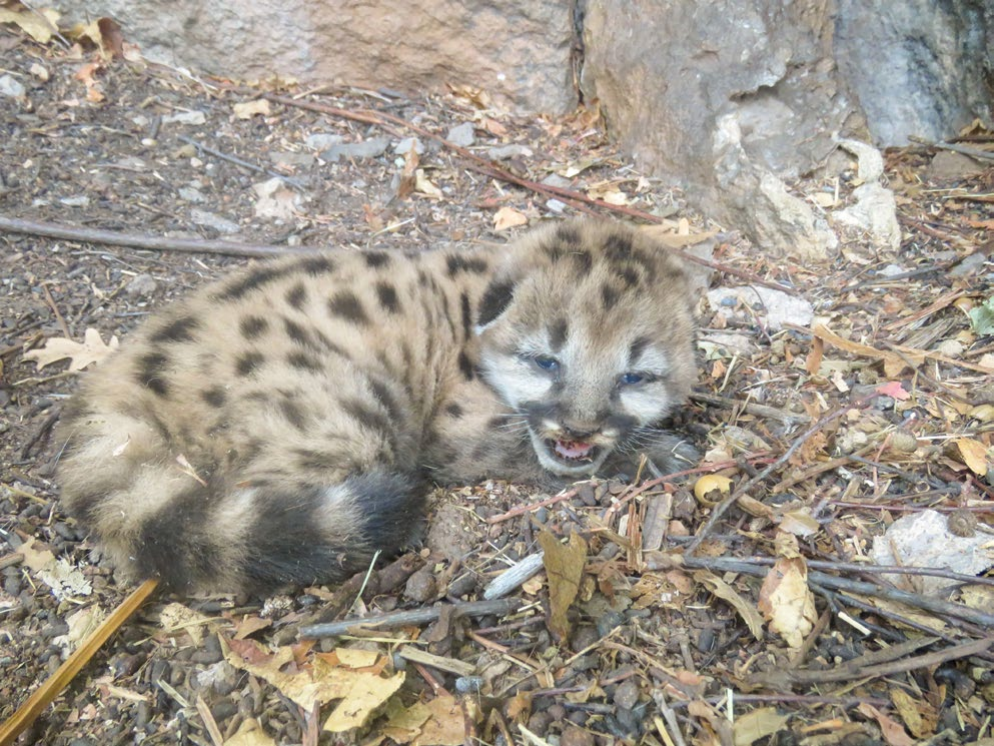
First identified litter for female 3 (F21) in the Jemez Mountains, New Mexico. A single ~2–3 week old kitten identified on 22 July 2018.

Female 3 (F21)—On 13 April 2018 we identified a collared male mountain lion (M10) sharing a kill with an uncollared female mountain lion. On 16 April 2018 we successfully captured the 45.4 kg, 4–5‐year‐old female. Between 9 and 21 July 2018 GPS data indicated that the female localized her movements consistent with denning. GPS data indicated that the female made a long, straight movement away from the suspected den at 2 am on 21 July 2018. On 22 July 2018 a researcher approached the identified site and found one kitten alive (Figure 4). The condition of the kitten indicated that it was young (< 2 weeks old); eyes were half open, legs were unsteady while standing. We identified evidence that the female was accompanied by a male at subsequent “GPS clusters” between 22 July and 30 July 2018. The female never returned to the den, and the kitten did not survive. We are unsure if the female originally had more than one kitten, which may have been killed by the male, or if the litter only consisted of one kitten. However, given the mother's apparent excellent physical condition, and documented litter sizes average 2.5 kittens (Logan and Sweanor 2001; Quigley and Hornocker 2010; Sunquist and Sunquist 2002) one or more kittens may have been killed by the arriving male. The loss of kittens and the continued presence of the male may explain the female's failure to return to the den.

**FIGURE 5:**
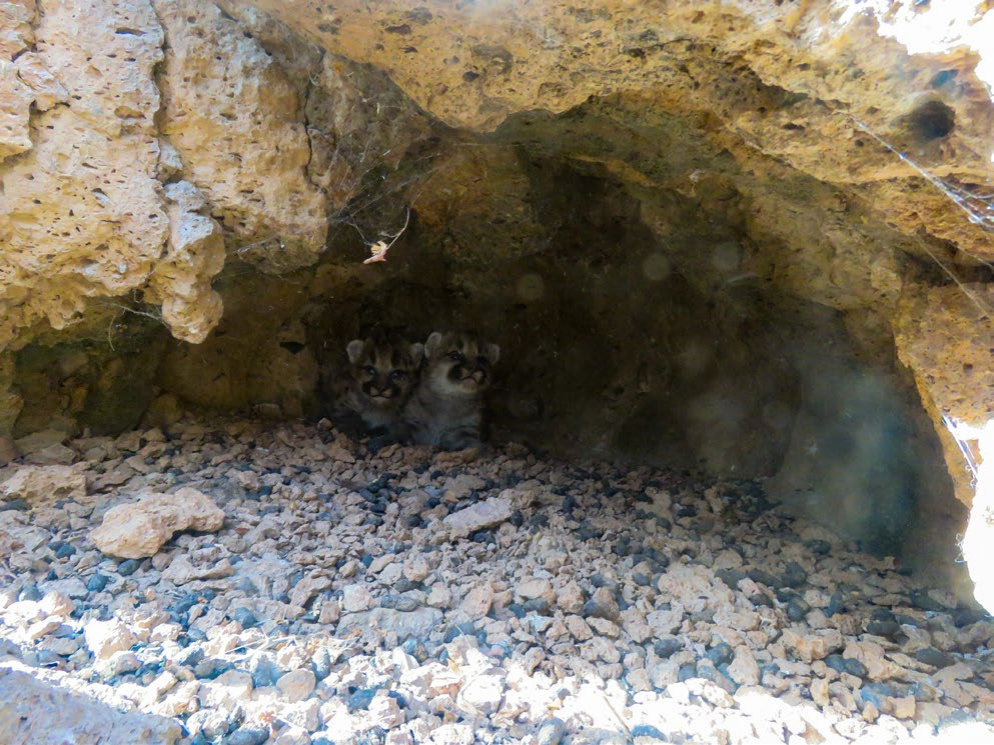
Second confirmed litter for female 3 (F21) in the Jemez Mountains, New Mexico. Three kittens confirmed on 20 November 2018.

Beginning 24 October 2018 GPS collar data again indicated that the female mountain lion began denning; 96 days after departing her previous kitten. Between evidence of this female traveling with a male within 24 h of “abandoning” her kitten, and the identified 96 days between denning, evidence indicates she successfully rebred in as few as 1–4 days after her previous litter. On 20 November 2018 one researcher approached the new den site while the female was away and identified three kittens. The condition of the kittens indicated that they were ~3–4 weeks old; eyes and ears were fully opened, legs were slightly unsteady but still capable of mobility (Figure 5). On 6 March 2019 a collared male (M19) usurped an elk kill from this female, and her collar failed on 9 March 2019. The fate of the kittens following the encounter with the male (M19) is unknown; however, this female was observed on camera trap multiple times in 2019, and no kittens were observed traveling with her. In March and April 2020 this female was again regularly observed on camera trap visiting communication scrape sites with no kittens present.

**FIGURE 6:**
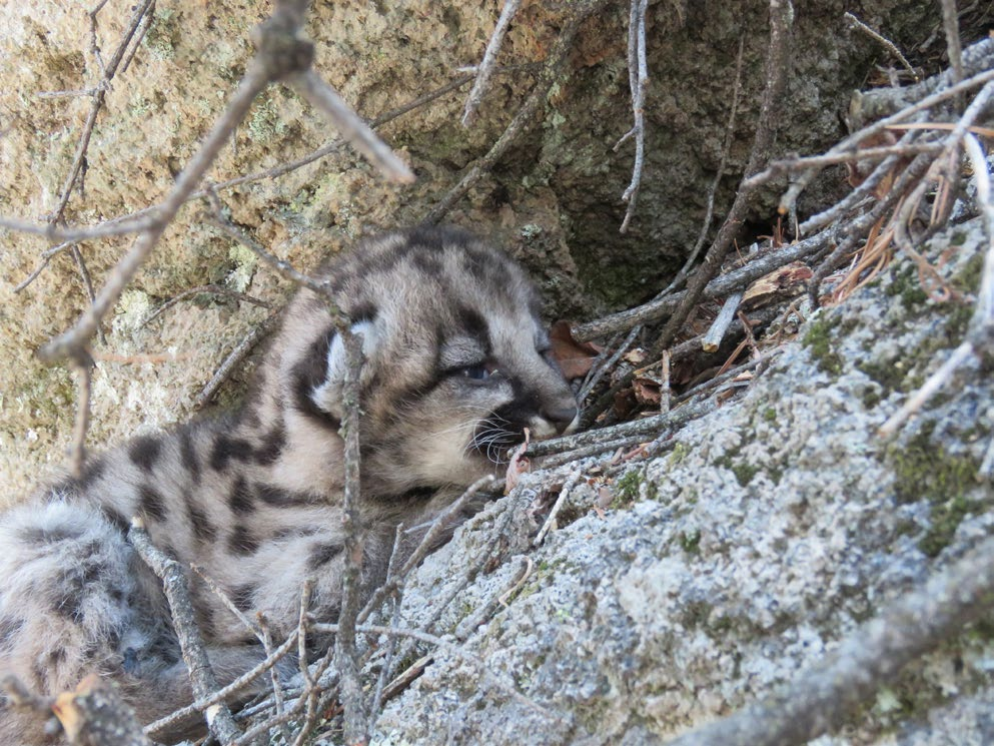
First confirmed litter for female 4 (F25) in the Jemez Mountains, New Mexico. A single ~2–3 week old kitten identified on 5 July 2021.

**FIGURE 7:**
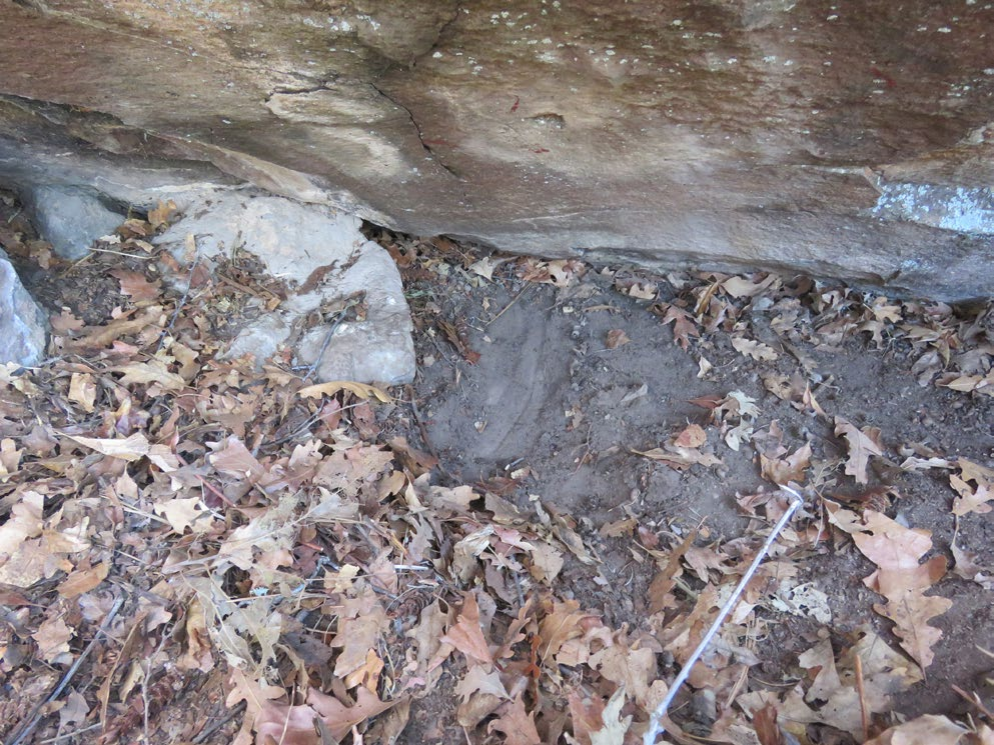
Confirmed den site for female 4 (F25), in the Jemez Mountains, New Mexico. Litter mortality confirmed on 7 April 2022. Note tracks and scrape inside the den and blood located on the rock directly above the scrape.

Female 4 (F25)—On 3 December 2020 we captured a 43.5 kg, 3–4‐year‐old female. There was no indication that she was pregnant or had kittens at the time of capture. On 16 March 2021 trail camera images indicate she was traveling with an uncollared male. On 21 June 2021, her GPS data indicated she initiated denning. On 2 July 2021 the female moved away from the suspected den and on 5 July 2021 we visited the site and confirmed 1 kitten located outside of the den, laying in a small crack of rock (Figure 6). The kittens' eyes were open, but mobility was very limited. GPS collar data indicates the female did not return to the site after leaving on 2 July 2021, and the kitten did not survive. It is unknown if the litter originally consisted of 1 kitten, or if 1 or more kittens may have been killed prior to our arrival; however, images from a camera trap at a mule deer kill site on 5 July 2021 show no signs of injury and no presence of other kittens or male mountain lions. On 11 July 2021 this female was observed visiting multiple communication scrape sites and vocalizing with a caterwaul; we were unable to confirm if an uncollared male was present with her. From 21 to 29 August 2021 the female was again observed traveling with an uncollared male. GPS collar data indicates she began denning on 25 November 2021; however, on 3 December 2021 she departed the den site and did not return. We visited the suspected den site on 11 December 2021 and found a protected boulder structure with litter disturbance and hair indicative of denning. Subsequent GPS data and camera trap monitoring of the adult female confirms that she was no longer caring for kittens. Unfortunately, we were unable to confirm the number and cause of death for these kittens, and, on 28 December 2021, this female was observed on camera trap images traveling with an uncollared male. On 12 March 2022 this female killed a large bull elk in an area outside of her normal home range. On 1 April 2022 the female again began denning in a rock outcropping ~80 m from her bull elk kill. On 5 April 2022 a GPS collared male mountain lion (M31) arrived to scavenge on the bull elk and also found the den site. The female promptly left the area upon arrival of the male, and we visited the den on 7 April 2022 and confirmed the kittens (number and sex unknown) were killed by the collared adult male mountain lion (Figure 7).

**FIGURE 8:**
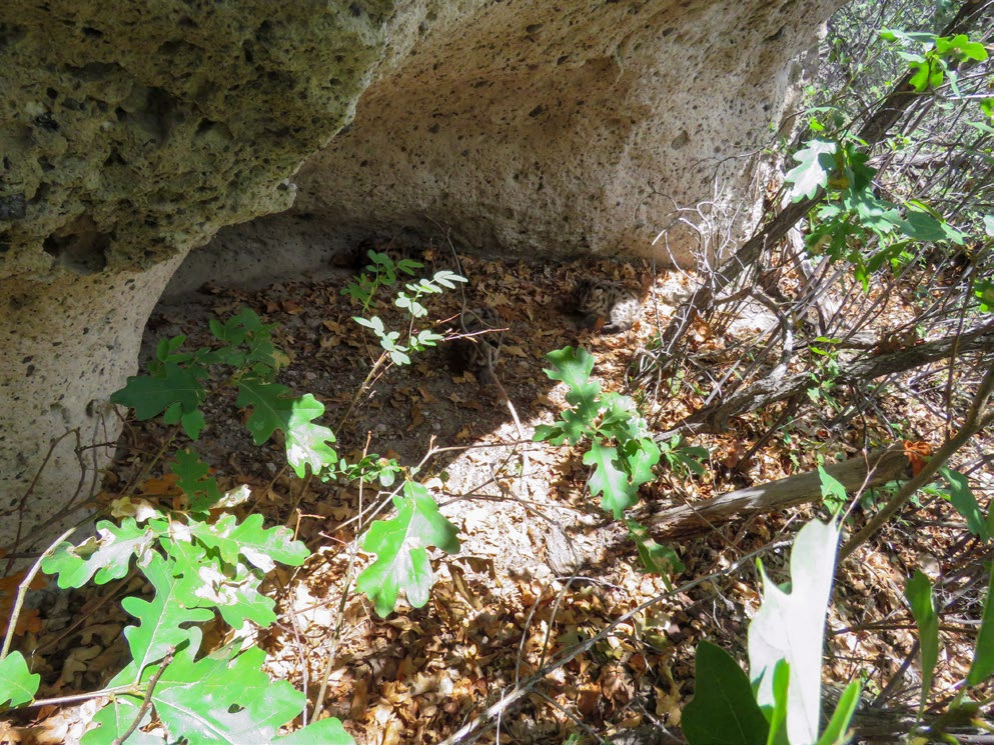
Confirmed litter of kittens for female 4 (F25) in the Jemez Mountains, New Mexico. Three kittens (2 male and 1 female) confirmed on 16 July 2022, born ~102 days after previous litter was killed by radio‐collared male mountain lion.

Between 15 – 17 April 2022 (10 days after losing kittens) the collared female was documented to be traveling with a different collared young ~2.5 years old male (M38). On 16 July 2022 the collared female again initiated denning (102 days after previous kittens were killed by a male mountain lion) indicating that the previous breeding event 10 days post‐kitten mortality was successful. On 6 August 2022, three kittens were confirmed and estimated to be ~3 weeks old (Figure 8). Of this litter, the female was successful in raising one kitten to ~12 months old before she again rebred and the kitten was forced to survive independently.

**FIGURE 9:**
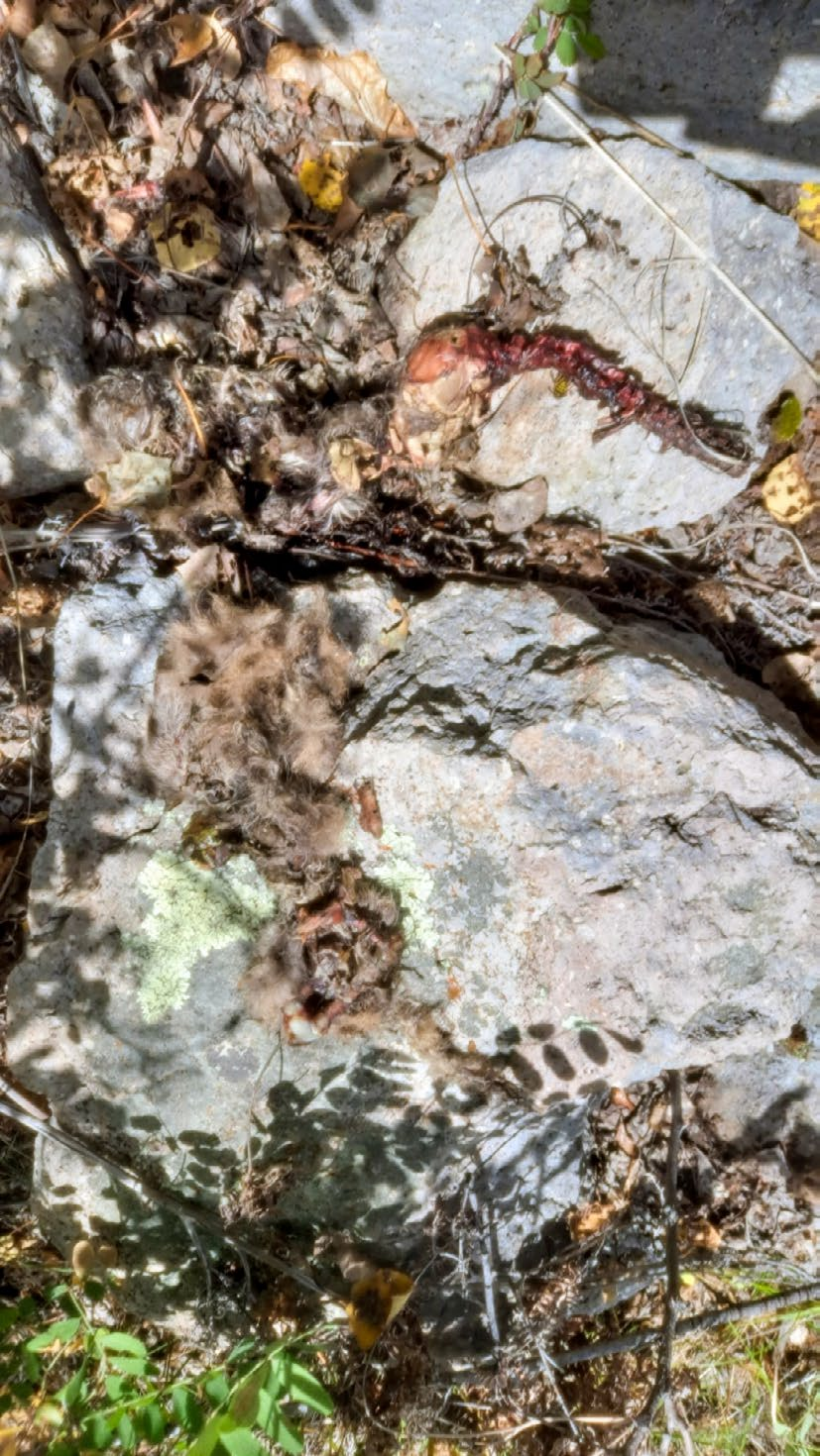
Confirmed litter loss for female 5 (F29) in the Jemez Mountains, New Mexico. Total number and sex of kittens were unknown, litter confirmed dead on 15 September 2022.

Female 5 (F29)—On 24 May 2021 we captured a 49.4 kg, 5–6 year old female. During the capture two kittens estimated to be ~4–5 months old were present at the site. The fate of these kittens is unknown, and the following summer, between 17 and 19 May 2022 the female was observed traveling with a radio collared male (M35) for ~3 days. On 30 August 2022, the female's collar data indicated localized movements and missing data points indicative of denning. On 11 September 2022, the female no longer showed localized movement and on 15 September 2022, we visited the clusters and identified one dead kitten we estimated to be less than 2 weeks old (Figure 9). The original number and sex of kittens at this den site could not be determined. On 25 September 2022 the female was observed on camera trap sharing a kill with an uncollared adult male for ~2 days. Between 12 and 20 December 2022 the female made atypical large movements outside of her traditional range; however, we were unable to access these sites to determine behavior.

On 20 March 2023 the female killed a bull elk and beginning 21 March 2023 she started forming a second cluster 381 m away from the kill, in a very secluded difficult to access rock outcropping. She continued moving between the sites until the evening of 5 April 2023. We visited the site on 14 April 2023 and found a site indicative of denning with a large rock overhang, thick obscuring vegetation and fur matted depression under the rock. Immediately outside the den, the surrounding area showed heavy vegetation disturbance, broken branches, and mountain lion hair. No presence of kittens was found, and movement data from F29 indicate that she likely lost the litter instead of moving kittens prior to our arrival. After leaving this site, the female killed a female elk 17 April 2023; a camera trap placed at the site identified an uncollared male at the site with the female. Movement data show indications of potential denning again beginning 3 September 2023. Unfortunately, we were unable to access this location to verify and beginning 29 September 2023 the female left the site with no evidence of kittens.

**FIGURE 10:**
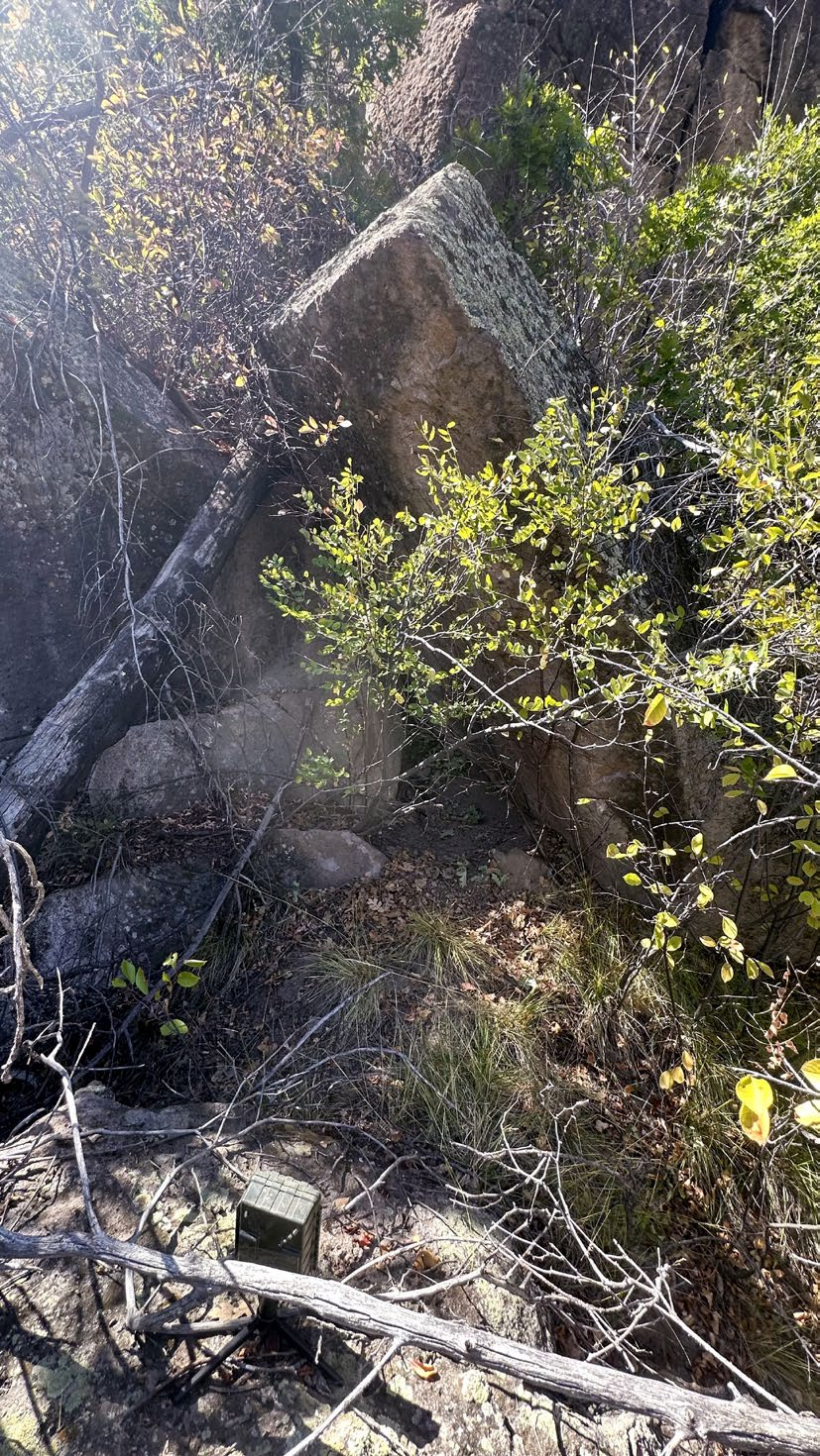
Photograph of confirmed female 6 (F43) den site with kittens in September 2023.

Female 6 (F43)—On 20 April 2023 we identified an uncollared female feeding on an elk kill with a collared male (M35). On 21 April 2023 we captured an ~45 kg, 7–9 year old female, and identified that she had three young kittens, estimated at ~2–3 months old (Video 2). A camera trap at this kill site also identified an additional uncollared mountain lion rotating feeding with this family group. Evidence from a GPS cluster on 26 April 2023 indicates that the kittens were traveling with the female; however, a video of this female on 13 May 2023 showed new injuries across the female's forearms and back, and no kittens were observed (Video 3). Subsequent photographs and videos of this female over the next 2 months showed no evidence of the kittens surviving. On 17 June 2023 we observed this female traveling with a collared adult male (M35) for ~2 days; 36 to 53 days following the loss of her kittens. GPS data indicates that the female began denning on 16 September 2023; however, camera trap images indicate that she continued to feed at a nearby kill while pregnant until 20 September 2023 before giving birth to a litter of three kittens (Figure 10).

**VIDEO 2:** Video of female 6 (F43) and her first confirmed litter of kittens sharing a kill site in April 2023. Video content can be viewed at https://onlinelibrary.wiley.com/doi/10.1002/ece3.73416.

**VIDEO 3:** Video of female 6 (F43) following suspected loss of litter in May 2023. Note abrasions on forearms and back. Video content can be viewed at https://onlinelibrary.wiley.com/doi/10.1002/ece3.73416.

## 4 Discussion

Our observations indicate that estrous cycles in female mountain lions can resume shortly after the loss of a litter, with successful breeding in our study area occurring ≤ 4–94 days following litter loss. Similar patterns have been reported elsewhere for mountain lions and other large cats (Table 2); most notably, cheetahs were reported to successfully rebreed as quickly as 2–5 days following litter mortality (Kelly et al. 1998; Laurenson 1994). This quick resumption of estrus after litter loss allows females to capitalize on reproductive opportunities, potentially serving as a compensatory mechanism to mitigate the fitness costs of litter loss. In Florida, Bartareau (2020) similarly documented a resumption of estrus in female mountain lions following unsuccessful litters, with females producing another litter 0.61–1.28 years later, suggesting a breeding lapse of approximately 0.35–1.02 years, although the cause of litter loss was not explicit. Earlier work by Logan and Sweanor (2001) in New Mexico, reported a mean interval of 100.4 ± 116.9 days (range: 23–307 days) between litter loss and subsequent breeding. Notably, the shortest interval observed by Logan and Sweanor (2001) followed the loss of stillborn kittens, suggesting that the nature and timing of litter loss may influence the duration before rebreeding. In contrast, females whose litters were lost to infanticide exhibited more variable intervals to rebreeding (e.g., 74 and 307 days following the death of kittens at 66 and 34 days of age, respectively). This variability in rebreeding intervals underscores the potential interplay between physiological readiness and environmental factors in shaping reproductive strategies in mountain lions and other large cats.

**TABLE 2:** Interbirth intervals (months) estimated by this study in Jemez Mountains, New Mexico from 2016 to 2024, in comparison with other mountain lion ( *Puma concolor* ) study areas and other selected big cat species: Leopard ( *Panthera pardus* ), cheetah ( *Acinonyx jubatus* ), jaguar ( *Panthera onca* ), lion ( *Panthera leo* ), and tiger ( *Panthera tigris* ).

	Interval between successful litters	Interval between unsuccessful litters
**Mountain lion (*Puma concolor*)**		
This study	19.8 ± 5.1 (*n* = 3) range 14.2–24.3	5.5 ± 1.9 (*n* = 10) range 3.5–9.4
Bartareau (2020)	26.8 ± 1.4 (*n* = 54) range 15.8–59.5	11.2 ± 0.4 (*n* = 40) range 7.3–15.4
Logan and Sweanor (2001)	17.4 ± 2.5 (*n* = 16) range 12.6–22.1	
Lindzey et al. (1994)	24.3 ± 6.8 (*n* = 7) range 19–40	13.7 ± 2.1 (*n* = 3) range 12–16
**Leopard (*Panthera pardus*)**		
Balme et al. (2013)	25 ± 1.0 (*n* = 55) range 14–39	11.0 ± 1.0 (*n* = 46) range 4–36
**Cheetah (*Acinonyx jubatus*)**		
Kelly et al. (1998)	20.1 (*n* = 36)	
**Jaguar (*Panthera onca*)**		
Fragoso et al. (2023)	21.8 ± 3.2 (*n* = 10) range 17–27	9.5 ± 4 (*n* = 4) range 6–15
**African Lion (*Panthera leo*)**		
Schaller (1972)	22.1 ± 4.1 (*n* = 7)	9.3 ± 4.3 (*n* = 13)
**Asiatic Lion (*Panthera leo persica*)**		
Banerjee and Jhala (2012)	27 ± 4.92 (*n* = 2)	12.2 ± 0.7 (*n* = 5)
**Bengal tiger (*Panthera tigris tigris*)**		
Singh et al. (2013)	33.4 ± 3.7 (*n* = 22) range 24–65	
Singh et al. (2013)	25.2 ± 1.8 (*n* = 18) range 19–32	6 ± 1.0 (*n* = 3) range 5–7
**Amur tiger (*Panthera tigris altaica*)**		
Kerley et al. (2003)	21.4 ± 4.4 (*n* = 7) range 14–39	7.0 ± 0.25 (*n* = 1)

*Note:* Adapted from Fragoso et al. (2023).

We observed multiple female counterstrategies attempting to avoid infanticide during our research including the use of multiple den sites, breeding with multiple males, and male avoidance (Palombit 2015). However, females were unable to eliminate the threat of infanticide. In at least one observed case in the Jemez Mountains, the perpetrator of infanticide was also the sire of the subsequent litter, highlighting the direct reproductive advantage of infanticidal behavior. By eliminating unrelated offspring, the male effectively advanced the female's estrous cycle, allowing him to sire a new litter sooner than if the original kittens had survived. This behavior aligns with sexually selected infanticide theory, in which males optimize their reproductive success by increasing mating opportunities (Ebensperger 1998; Hrdy 1979). Conversely, in other cases, the perpetrator of infanticide was not the sire of the subsequent litter, failing to directly capitalize on sexual selection through infanticide; however, their actions may still prove beneficial by reducing competition and providing additional nutrition (Palombit 2015).

In several cases, we observed that females who lost their litters were denning near a kill site, though it remains unclear whether this proximity contributed to the mortality event. However, it is well documented that carcasses serve as focal points for carnivore aggregation, increasing encounter rates among conspecifics and competitors (Elbroch, Levy, et al. 2017; Prugh and Sivy 2020). In our study system, our observations of black bear predation on kittens and of M31's killing of female 4's (F25) kittens were both suggestive that carrion may have first attracted the dominant competitors, which then killed the vulnerable kittens. Similar observations have been reported in jaguars, where large livestock carcasses attracted overlapping jaguars resulting in documented infanticide events (Tortato et al. 2017). Such observations suggest that spatial and temporal factors, including den selection and overlapping with kill sites, may elevate the risk of infanticide or other forms of mortality. Despite the potential risk, denning near a kill may reflect trade‐offs in maternal behavior, where females prioritize access to prey thereby reducing travel time and associated energetic costs, and increasing feeding and maternal care (Engebretsen et al. 2024). Understanding these dynamics is crucial, as the location of denning sites and the timing of estrus cycles have implications for maternal fitness, kitten survival, and overall population dynamics in mountain lion populations. Further research is needed to disentangle these contributing factors and their impact on reproductive strategies.

We observed two cases of litter abandonment in the Jemez Mountains. In both of our observed cases, food for the adult female did not appear limiting, as both were making frequent ungulate kills and appeared to be in excellent physical condition. In the case of female 3 (F21), we confirmed that she was traveling with a male within 24 h of the kitten abandonment. Despite being unable to document if the male contributed to the original abandonment (i.e., partial litter predation or persistent harassment of the female), the temporal and spatial proximity is suggestive of his involvement. Although female mountain lions are known to use the strategy of exhibiting pseudo‐estrus behavior when nursing young kittens in attempts of avoiding infanticide from an unknown male (Benson et al. 2012), in both of our observations the females failed to return and the kittens subsequently died. There have been reports of other female carnivores, notably lions and grizzly bear ( *Ursus arctos horribilis* ), abandoning their litters when only a single young cub remains (Packer and Pusey 2008). It was suggested that when the average litter size of a female is ≥ 2 offspring, abandonment of a litter of one would allow the female to increase her reproductive success by abandoning the current young offspring in favor of a subsequent larger litter size (Bateson 1994; Tait 1980). However, such a strategy is likely highly context dependent and influenced by life history traits, maternal age and condition, and ecological pressures (Mendl 1994). For example, leopards have been documented maintaining single‐offspring litters and extending maternal care during periods of reduced prey density (Balme et al. 2017). In both abandonment cases we observed, quick rebreeding following the abandonment of the single kitten did occur, supporting the concept of improved reproductive success. Despite these observations, we are limited in our interpretation of why the abandonment occurred. For example, it is possible that the females perceived the litter as already lost (i.e., if partial litter predation occurred), or other undocumented factors such as perceived risk to the female, disturbance, stress, maternal physiology, or disease may have played a role (Clark et al. 2015; Doran‐Myers et al. 2025; Hrdy 1979; Laurenson 1994).

The cases of female 1 (F11) and female 2 (F16) from the Jemez Mountains study site highlights the physiological toll of maternal care and the challenges of maintaining adequate nutrition during periods of high energetic demand. Following the loss of their first litters, and during the rearing of their second recorded litters, both females exhibited a marked reduction in body mass. Female 1 (F11) relied exclusively on smaller prey species from areas surrounding a human residential area, including feral house cats (*Felis silvestris catus*), raccoons ( *Procyon lotor* ), and rabbits (*Lepus* spp.), suggesting that she was unable to secure larger prey during this critical period. The reliance on smaller prey is likely influenced by multiple factors, including the limited hunting range associated with maternal denning, and the availability of prey. Similarly, in Africa, cheetahs with young kittens were found to be operating at an energetic deficit and were behaviorally constrained by the den location, resulting in reduced hunting ranges and differences in hunting behavior (Laurenson 1995). Lactating female mountain lions require ~2.3 kg of meat per day to meet their energetic needs (Laundre 2009); as such, this shift in diet to small prey likely resulted in insufficient caloric intake to support both her own energy needs and those of her kitten requiring her to rely on stored body fat, reducing her overall body mass (Heldstab et al. 2017). Utilizing human residential areas may have yielded a consistent small prey food source or provided perceived safety from other large carnivores; however, it also dramatically increased the risk of human‐carnivore conflict. While smaller prey species may prove easier to capture, they also provide significantly less caloric value than large ungulates, thereby requiring increased hunting frequency and effort, potentially contributing to the loss of the litter (Eloff 1980; Maehr et al. 1989). Following the loss of her second litter, female 1's (F11) movement patterns expanded, allowing her to target larger prey species such as ungulates. This behavioral shift suggests that the constraints of maternal care were limiting her foraging efficiency and prey selection during the period of kitten maintenance (Laurenson 1995; Maehr et al. 1989). Similarly, female 2 (F16) displayed a marked reduction in body condition following the loss of her litter. It was ~2 weeks following female 2 (F16) rebreeding that we documented the only case of probable female infanticide in our study with female 2 (F16) locating and consuming female 1's (F11) kitten. Although infanticide among large cats is most commonly identified as a form of sexual selection whereby males exert pressure on females, as was identified in leopards (Balme and Hunter 2013), female to female infanticide has been widely documented and may represent a strategy for resource exploitation (e.g., predation) or a form of reducing future resource competition (Ebensperger 1998; Hrdy 1979). This case highlights the potential tradeoffs female mountain lions navigate when nutritional limitations, energetic demands, and the risks of predation or infanticide converge to influence reproductive success and survival (Engebretsen et al. 2024; Kelly et al. 1998; Laurenson 1994, 1995; Maehr et al. 1989).

Our observations have important implications for the management and monitoring of mountain lion populations. Accounting for the potential for rapid kitten loss and subsequent rebreeding when assessing population dynamics and reproductive rates would improve management. In particular, the physiological toll on females following litter loss and rebreeding must be considered, as repeated cycles of gestation and lactation can significantly deplete body condition, potentially reducing maternal survival and subsequent reproductive success. This strain is exacerbated when females are forced to subsist on smaller prey due to limited availability of large ungulates, further compounding the energetic costs of reproduction. Additionally, litter loss can disrupt the seasonal timing of births, leading to kittens being born during suboptimal periods, such as winter, with reduced prey availability (Jansen and Jenks 2012). Prey availability has been shown to be an important factor impacting the growth and survival of kittens, ultimately influencing population dynamics (Jansen and Jenks 2012; Laundre et al. 2010). Additionally, kittens born outside of the ungulate birth peak were shown to disperse at a younger age and with a smaller body size, potentially reducing their competitiveness due to limited experience hunting and securing prey (Elbroch, Feltner, and Quigley 2017; Jansen and Jenks 2012). Although not analyzed here, these impacts may be more prevalent in territorial unstable local populations and areas with frequent transient males, contributing to an increased prevalence in infanticidal behavior (Keehner et al. 2015; Leclerc et al. 2017; Ruth et al. 2011). Addressing these factors is crucial for effective population management and conservation strategies.

For field researchers, our results highlight the need to refine methods for identifying and monitoring denning activities. Traditional techniques, such as analyzing long‐duration GPS clusters where females return over multiple weeks, may fail to detect dens lost to infanticide or interspecific competition. This underestimation could skew data on reproductive success and kitten survival. Additionally, identification and long‐term monitoring of community “scrape” sites using camera traps can assist in documenting otherwise difficult to detect reproductive behaviors. Combining field visitations and camera trap monitoring with mountain lion behavioral cues and GPS collar data would likely improve detection of denning events. These adjustments will enhance our understanding of reproductive ecology and support more effective conservation strategies for mountain lion populations.

## Author Contributions

**Mark A. Peyton:** investigation (equal), writing – original draft (equal), writing – review and editing (equal). **Brian D. Jansen:** conceptualization (equal), investigation (equal), writing – original draft (equal), writing – review and editing (equal). **James W. Cain III:** funding acquisition (equal), project administration (equal), supervision (equal), writing – original draft (equal), writing – review and editing (equal). **Jonathan A. Jenks:** conceptualization (equal), funding acquisition (equal), project administration (equal), writing – original draft (equal), writing – review and editing (equal).

## Funding

This work was supported by South Dakota Game, Fish and Parks, W‐75‐R (Study Number 7587); U.S. Geological Survey; U.S. Forest Service; National Park Service.

## Conflicts of Interest

The authors declare no conflicts of interest.

## Data Availability

Data collected for this project is available in Tables 1 and 2 of this manuscript.
